# Reproductive outcomes of ectopic pregnancy with conservative and surgical treatment: A systematic review and meta-analysis

**DOI:** 10.1097/MD.0000000000033621

**Published:** 2023-04-28

**Authors:** Hong-Juan Hao, Li Feng, Li-Fei Dong, Wei Zhang, Xiao-Li Zhao

**Affiliations:** a Department of Gynecology, The Fourth Hospital of Shijiazhuang, Shijiazhuang, Hebei, China.

**Keywords:** ectopic pregnancy, expectant treatment, fertility, intrauterine pregnancy, MTX, repeat ectopic pregnancy, salpingectomy, salpingostomy

## Abstract

**Background:**

Ectopic pregnancy (EP), one of the most common gynecological emergencies, is the major cause of maternal death in the first trimester and increases the incidence of infertility and repeat ectopic pregnancy (REP). The aim of this study was to compare the effects of different treatment methods for tubal EP on natural pregnancy outcomes.

**Methods:**

We systematically searched PubMed, Embase, Cochrane Library, Web of Science, and Clinical Trials for observational studies on EP (published until October 30,2022 in English) comparing methotrexate (MTX) versus surgery, MTX versus salpingostomy, MTX versus salpingectomy, salpingostomy versus salpingectomy, and MTX versus expectant treatment. Our main endpoints included subsequent natural intrauterine pregnancy (IUP) and REP. We assessed the pooled data using Review Manager software (version 5.3) with a random effects model.

**Results:**

Of 1274 identified articles, 20 were eligible and 3530 participants were included in our analysis. There was a significant difference in the odds of subsequent IUP in tubal EP patients who underwent MTX compared with those who were treated with surgery [odds ratios (OR) = 1.52, 95% confidence interval (CI):1.20–1.92]. No significant difference was found in the odds of REP between the 2 groups (OR = 1.12, 95% confidence interval [CI]: 0.84–1.51). There was no significant difference in the odds of subsequent IUP and REP in patients after MTX compared to those after salpingostomy (OR = 1.04,95% CI: 0.79–1.38; OR = 1.10, 95% CI: 0.64–1.90). There was a significant difference in the odds of subsequent IUP in patients after MTX compared with those after salpingectomy (OR = 2.11, 95% CI: 1.52–2.93). No significant difference was found in the odds of REP between the 2 groups (OR = 0.98, 95% CI: 0.57–1.71). There was a significant difference in the odds of subsequent IUP between patients who underwent salpingostomy and those who underwent salpingectomy (OR = 1.61, 95% CI: 1.29–2.01). No significant difference was found in the odds of REP between the 2 groups (OR = 1.21, 95% CI: 0.62–2.37). There was no significant difference in the odds of subsequent IUP and REP in patients after MTX compared with those after expectant treatment (OR = 1.25, 95% CI: 0.64–2.45; OR = 0.69, 95% CI: 0.09–5.55).

**Conclusion:**

For hemodynamically stable tubal EP patients, MTX has advantages over surgery, particularly salpingectomy, in improving natural pregnancy outcomes. However, MTX is not inferior to salpingostomy and expectant treatment.

## 1. Introduction

Ectopic pregnancy (EP) remains one of the most common gynecological emergencies and is a leading cause of maternal death in early pregnancy,^[1]^ affecting 1% to 2% of all pregnant women^[2]^ and increasing infertility and repeat ectopic pregnancy (REP).^[3]^ EP is defined as pregnancy outside the uterine cavity, mostly occurring in the fallopian tube (96%).^[4]^ Surgical treatment is considered the gold standard. With advances in early diagnosis, such as human chorionic gonadotropin (HCG) levels and transvaginal ultrasound,^[5,6]^ EP can be diagnosed in the early stage, significantly reducing the emergency operation rate and mortality rate of EP and enabling several patients to choose expectant treatment or medical treatment, such as methotrexate (MTX). Naveed et al^[7]^ found that expectant treatment was as safe and effective as MTX in patients with EP with stable hemodynamic conditions and decreased or low HCG levels. The overall success rate of MTX has been reported to be as high as 90% for the appropriate indications.^[8,9]^ There is increasing attention to reproductive outcomes for EP patients, especially for those who want to have a child, so it is crucial to clarify the impact of each treatment modality on natural pregnancy outcomes in order to help tubal EP patients with fertility requirements choose an appropriate treatment.

We reviewed the literature on reproductive outcomes after conservative and surgical interventions. The primary outcome indicators were subsequent intrauterine pregnancy (IUP) and REP. The objective of this study was to investigate the natural pregnancy outcomes by comparing MTX versus surgery, MTX versus salpingostomy, MTX versus salpingectomy, salpingostomy versus salpingectomy, and MTX versus expectant treatment.

## 2. Materials and methods

This meta-analysis was written in accordance with the PRISMA statement and registered on INPLASY, the registration number: DOI 10.37766/inplasy202320032.

### 2.1. Study selection

In this study, Patients with EP met the following inclusion criteria: A diagnosis of tubal EP by ultrasound; Stable hemodynamics; Treated with expectant treatment, MTX or salpingostomy, salpingectomy; Women of childbearing age with natural fertility requirements, and; IUP and REP as the primary outcome indicators. The exclusion criteria were as follows: Non-EP, such as ovarian pregnancy or cervical pregnancy; Rupture of the fallopian tube indicated by ultrasound; Treatment except expectant treatment, MTX or salpingostomy, and salpingectomy; Those with no reproductive requirements or those planned to receive assisted reproductive technology, and; Case reports, reviews, original studies with incomplete data and low-quality research.

### 2.2. Data sources

We electronically searched PubMed, Embase, Cochrane Library, Web of Science, and Clinical Trials for observational studies (published until October 30, 2022 in English). All articles on conservative and surgical treatment for tubal EP were retrieved. Medical subject headings and free text words included “EP*, Pregnancies, Ectopic, Extrauterine Pregnancy*, Amethopterin, Mexate, Methotrexate*, Dicesium Salt Methotrexate, Salpingectom*, Tubectom*, Tubal Excision*, Salpingostom*”. References of the original and reviewed articles were manually searched, and the relevant literature was included.

### 2.3. Screening process

Two authors (H.J.H. and L.F.) independently screened all documents, which were then de-duplicated. The titles and abstracts of each article were screened separately by the 2 authors. The full texts were viewed according to the inclusion and exclusion criteria, except those that did not meet the inclusion criteria. Any disagreements between the 2 authors were resolved by a third author (L.F. D.).

### 2.4. Data extraction

Data extraction was performed separately by 2 authors (H.J.H. and L.F.) and any discrepancies were resolved by a third author (X.L.Z.). The following information was extracted: Basic information of the literature: title, author, date of publication, and country of publication; Basic characteristics of the study: study type, age, number of study subjects, treatment mode of EP, and years of follow-up; Primary outcome measures: IUP and REP, and; Quality assessment and bias risk assessment.

### 2.5. Literature quality evaluation

The Newcastle-Ottawa Scale (NOS) scales applied to observational studies were used for the quality evaluation. The quality evaluation scale includes 8 items in 3 categories. In the categories “selection” and “exposure,” the quality evaluator gave at most 1 star to each item, except for 2 stars for comparative items, with a total score of 9 points. Each study was scored separately, and scores ranged from 0 to 9. For each study, assessments were performed separately by 2 authors (X.L.Z. and W.Z.), and discrepancies were resolved in consultation with a third author (H.J.H.).

### 2.6. Statistical analysis

The reproductive outcome measures assessed were IUP and REP, which were dichotomous variables, and the data were expressed as odds ratios (OR) and 95% confidential interval (CI). We used a random effects model (D-L method) combined with OR values and drew forest maps and funnel plots for each study. The *I*^2^ statistic was used to assess statistical heterogeneity. *I*^2^ > 50%signified moderate-to-high heterogeneity. Statistical analysis was performed using the Review Manager software (version5.3).

## 3. Results

**Figure 1.**
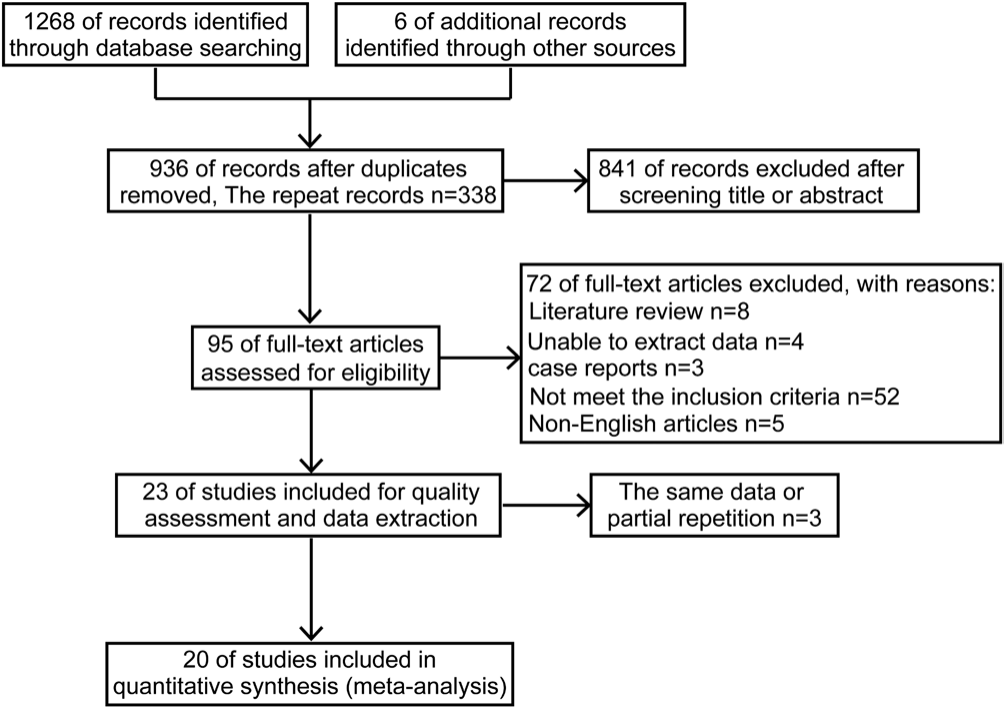
Flow chart of literature screening.

A total of 1274 articles were initially retrieved by electronic searches, including 338 duplicate references. Of the remaining 936 articles, 95 were included after screening titles and abstracts, excluding unrelated articles, review articles, meeting abstracts, case reports, case series, personal views, and editorials. After a full text browsing of the remaining 95 articles, 20 were screened for final inclusion. Of these, the full text of 1 article was not retrieved, but as a randomized controlled trial (RCT) study, it was included because of complete data. This study was confined to trials published in English. A screening flow chart is shown in Figure 1.

### 3.1. Study characteristics

The characteristics of the 20 included studies are listed in Table 1. All of these studies were published between 1999 and 2022, including 4 RCT trials, 10 retrospective cohort studies, 5 prospective study cohort studies and 1 cross-sectional study, with a sample size ranging from 36 to 1026 female patients, with a total number of 3530. Among the 20 included studies, the age of the sample population was 15 to 44 years, reproductive outcomes were compared between MTX and surgery in 20 studies, MTX and salpingostomy in 8 studies, MTX and salpingectomy in 10 studies, salpingostomy and salpingectomy in 6 studies, and MTX and expectant treatment in 5 studies. The follow-up period ranged from 1 year to 15 years. The NOS results are presented in Table 2. All studies had good results in the NOS, with scores ranging from 6 to 8 points. In this study, we combined the IUP and REP data of MTX versus surgery, MTX versus salpingostomy, MTX versus salpingectomy, salpingostomy versus salpingectomy, and MTX versus expectant treatment.

**Table 1:** Basic characteristics of 20 studies.

Study	Country	Study design	Age of study	Study population	Treatment methods	Duration of follow-up (Y)	Outcomes (IUP)	Outcomes (REP)
A. Al Toqi 2021^[10]^	Oman	RT	--	54 patients 72 patients	MTX or surgery	4–6	42/54 52/72	6/54 7/72
A.Daniilidis 2016^[11]^	Greece	RT	--	13 patients 23 patients	MTX or surgery2	1	8/13 12/23	1/13 1/23
Alla V. Boychuk 2020^[12]^	Ukraine	PT	26–30	20 patients 40 patients	MTX or surgery (1or2)	5–15	15/20 18/40	--
Azadeh Yousefnezhad 2018^[13]^	Iran	CS	28.56 ± 5.63	93 patients 21 patients	MTX or surgery	3	61/93 10/21	7/93 2/21
E.Demirdag 2016^[14]^	Ireland	RT	22–39	53 patients 32 patients	MTX or surgery	2–6	31/53	0 1/32
Gabriel Levin 2022^[15]^	Israel	RT	28–36	64patients 61 patients	MTX or surgery2	10	49/64 26/61	--
G Dias Pereira 1999^[16]^	Amsterdam	RCT	--	34 patients 40 patients	MTX or surgery1	1.5	12/34 16/40	3/34 4/40
Herve´ Fernandez 2013^[17]^	France	RCT	28–31	87 patients 211 patients	MTX or surgery (1or2)	4–9	58/87 144/211	8/87 16/211
J.Bouyer 2000^[18]^	France	PT	15–44	25 patients 266 patients	MTX or surgery(1or2)	2	20/25 178/266	3/25 27/266
JAN I. OLOFSSON 2001^[19]^	Sweden	RT	30 ± 6 32 ± 5	22 patients 35 patients	MTX or surgery	3–5	14/22 18/35	--
LARS BO KRAG MOELLER 2009^[20]^	Denmark	RCT	21.1–40.9 24.3–41.2	52 patients 52 patients	MTX or surgery	6.9–10.1	38/52 32/52	5/52 9/52
M.T. El-Sherbiny 2003^[21]^	--	RCT		58 patients	MTX or surgery	1	16/26 19/32	--
Marianne de Bennetot 2012^[22]^	France	PT	15–44	109 patients 917 patients	MTX or surgery (1or2)	2	83/109 670/917	21/109 123/917
N.Dalkalitsis 2006^[23]^	Greece	RT	19–44	28 patients 69 patients	MTX or surgery1	10	23/28 57/69	4/28 7/69
Senem Arda düz 2021^[24]^	Turkey	PT	18–45	42 patients 40 patients	MTX or surgery	2	35/42 35/40	--
Silvia Baggio 2021^[25]^	Italy	RT	36 ± 6 38 ± 5	38 patients 86 patients	MTX or surgery	1–6.5	21/38 34/86	4/38 7/86
Volkan Turan 2011^[26]^	Turkey	RT	18–28	29 patients 90patients	MTX or surgery (1or2)	2	20/29 56/90	3/29 8/90
Wassan R. Alkhafajy 2018^[27]^	Iraq	PT	--	110 patients 150 patients	MTX or surgery2	1	90/110 88/150	--
Yu-ting Shen 2022^[28]^	China	RT	30 ± 4	60 patients	MTX or surgery2	5	54/60 116/140	4/60 21/140
Zahra Asgari 2021^[29]^	Iran	RT	31.9 ± 5.6	64 patients	MTX or surgery (1or2)	1.5	22/64 36/130	6/64 10/130

CS = cross-sectional study, IUP = intrauterine pregnancy, MTX = methotrexate, PC = prospective, RC = retrospective, RCT = randomized controlled trial, REP = repeat ectopic pregnancy, surgery1 = salpingostomy, surgery2 = salpingectomy, Y = year.

**Table 2:** Appraisal of methodological quality (Newcattle-Ottawa Scale)of 20 studies.

Study	Case-cohort representative	Ascertainment of exposure	Outcome negative at start	Comparability by design	Outcome assessment	Duration of follow-up	Adequacy of follow-up	Score
Al Toqi 2021^[10]^	*	*	*	*	*	*	*	7
A.Daniilidis 2016^[11]^	*	*	*	*	*	†	*	6
Alla V.Boychuk 2020^[12]^	*	*	*	*	*	*	*	7
Azadeh Yousefnezhad 2018^[13]^	*	*	*	*	*	*	*	7
E.Demirdag 2016^[14]^	*	*	*	*	*	*	*	7
Gabriel Levin 2022^[15]^	*	*	*	*	*	*	*	7
G Dias Pereira 1999^[16]^	*	*	*	‡	*	*	*	8
Herve’ Fernandez 2013^[17]^	*	*	*	‡	*	*	*	8
J.Bouyer 2000^[18]^	*	*	*	*	*	*	*	7
Jan I. Olofsson 2001^[19]^	*	*	*	*	*	*	*	7
Lars Bo Krag^[20]^	*	*	*	‡	*	*	*	8
M.T. El-Sherbiny 2003^[21]^	*	*	*	‡	*	†	*	7
Marianne de Bennetot 2012^[22]^	*	*	*	*	*	*	*	7
N.Dalkalitsis 2006^[23]^	*	*	*	*	*	*	*	7
Senem Arda düz 2021^[24]^	*	*	*	*	*	*	*	7
Silvia Baggio 2021^[25]^	*	*	*	*	*	*	*	7
Volkan Turan 2011^[26]^	*	*	*	*	*	*	*	7
Wassan R. Alkhafajy 2018^[27]^	*	*	*	*	*	†	*	6
Yu-ting Shen2022^[28]^	*	*	*	*	*	*	*	7
Zahra Asgari 2021^[29]^	*	*	*	*	*	*	*	7

* Indicates that a feature is present.

† Indicates that a feature is absent.

However for comparability by design this checklist awards the maximum of 2 stars (

‡ ).

### 3.2. Reproductive outcomes after MTX versus surgery

**Figure 2.**
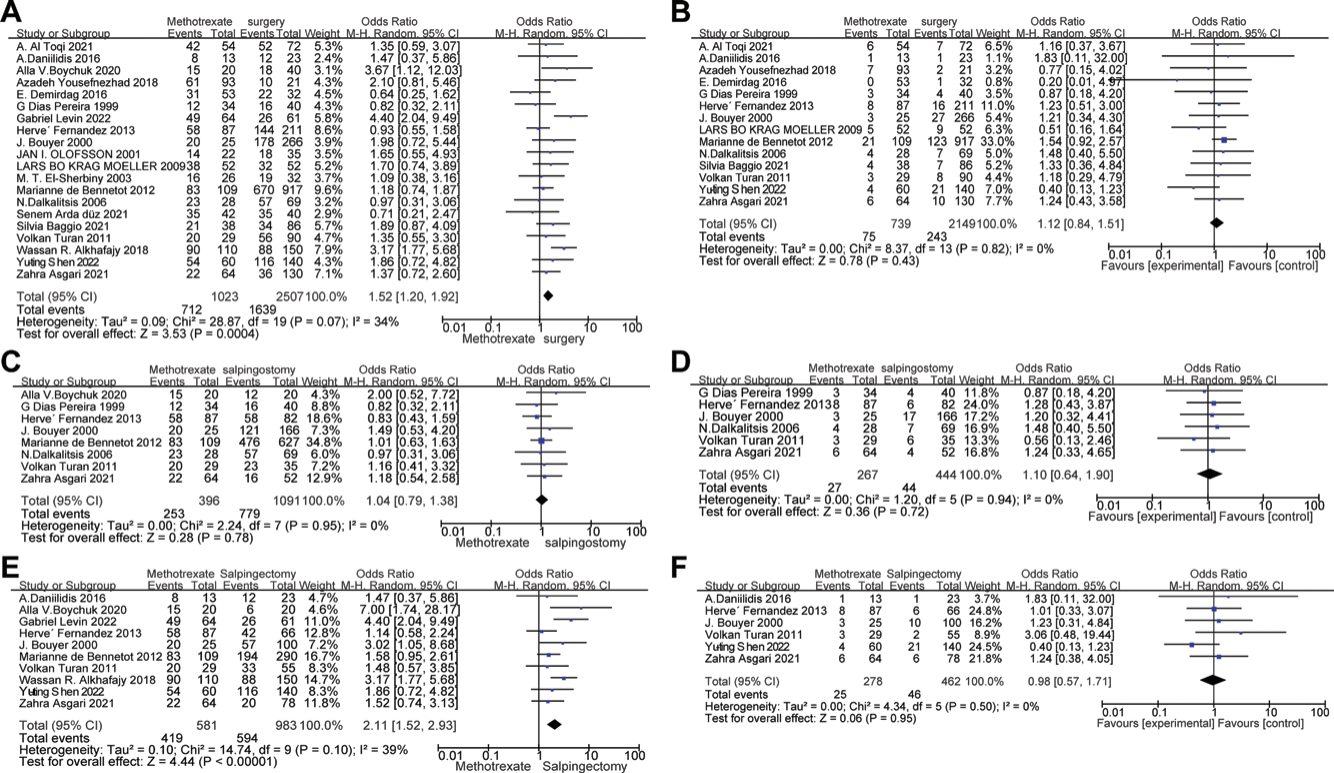
Forest plots of meta-analysis of IUP and REP in tubal EP. (A and B) Forest plots of combined IUP and REP after MTX and surgery, (C and D) Forest plots of combined IUP and REP after MTX and salpingostomy, and (E and F) forest plots of combined IUP and REP after MTX and salpingectomy. EP = ectopic pregnancy, MTX = methotrexate, REP = repeat ectopic pregnancy.

Twenty articles, including 3530 participants, compared reproductive outcomes after MTX with those after surgery. The characteristics, IUP and REP of the 20 articles are summarized in Table 1. There was a significant difference in the odds of subsequent IUP in patients who underwent MTX compared with those who were treated with surgery (OR = 1.52, 95% CI: 1.20 to 1.92;*P* < .001), and *I*^2^ = 34%, suggesting low heterogeneity. Of these, 14 articles summarized postoperative REP. There was no significant difference in the odds of subsequent REP, with a pooled OR of 1.12 (95% CI:0.84 to 1.51, *P* = .43), and *I*^2^ = 0%, suggesting that there was no significant heterogeneity in14 articles. The forest map is shown in Figure 2.

### 3.3. Reproductive outcomes after MTX versus salpingostomy

Eight articles involving 1487 people compared reproductive outcomes after MTX and salpingostomy. The characteristics of the literature and data are summarized in Table 1. There was no significant difference in the odds of subsequent IUP between patients who underwent MTX and those who were treated with salpingostomy (OR: 1.04, 95% CI: 0.79 to 1.38; *P* = .78), and *I*^2^ = 0%, suggesting good homogeneity. Among these, 6 articles summarized postoperative REP. There was no significant difference in the odds of subsequent REP, with a pooled OR of 1.10 (95% CI: 0.64 to 1.90, *P* = .72), and *I*^2^ = 0%, suggesting that the 6 articles had good homogeneity. The forest map is shown in Figure 2.

### 3.4. Reproductive outcomes after MTX versus salpingectomy

Ten publications involving 1564 participants compared reproductive outcomes after MTX and salpingectomy. The characteristics, IUP and REP of each study are summarized in Table 1. There was a significant difference in the odds of subsequent IUP in patients who underwent MTX and those who were treated with salpingectomy, with a pooled OR of 2.11 (95% CI: 1.52 to 2.93, *P* < .001) and *I*^2^ = 39%, suggesting slight heterogeneity. Among them, 6 articles summarized REP. There was no significant difference in the odds of subsequent REP, with a pooled OR of 0.98 (95% CI: 0.57 to 1.71, *P* = .95), and *I*^2^ = 0%, suggesting that the 6 articles had good homogeneity. The forest map is shown in Figure 2.

### 3.5. Reproductive outcomes after salpingostomy versus salpingectomy

**Figure 3.**
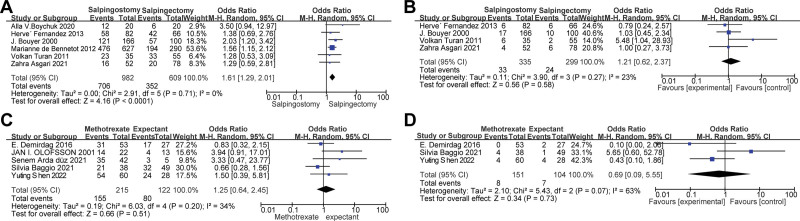
Forest plots of meta-analysis of IUP and REP in tubal EP. (A and B) forest plots of combined IUP and REP after salpingostomy and salpingectomy, (C and D) forest plots of combined IUP and REP after MTX and expectant treatment. EP = ectopic pregnancy, MTX = methotrexate, REP = repeat ectopic pregnancy.

Six publications involving 1591 participants compared reproductive outcomes after salpingostomy and salpingectomy. The characteristics, IUP and REP of each study are summarized in Table 1. There was a significant difference in the odds of subsequent IUP in patients who underwent salpingostomy and those who were treated with salpingectomy, with a pooled OR of 1.61 (95% CI: 1.29 to 2.01, *P* < .001) and *I*^2^ = 0%, suggesting good homogeneity. Among them, 4 articles summarized REP. No significant difference was found in the odds of subsequent REP, with a pooled OR of 1.21 (95% CI: 0.62 to 2.37, *P* = .58) and *I*^2^ = 23%, suggesting low heterogeneity. The forest map is shown in Figure 3.

### 3.6. Reproductive outcomes after MTX versus expectant treatment

Five publications involving 337 participants compared reproductive outcomes after MTX and expectant treatment. The characteristics, IUP and REP of each study are summarized in Table 1. There was no significant difference in the odds of subsequent IUP in patients who underwent MTX compared with those who were treated with expectant treatment, with a pooled OR of 1.25 (95% CI: 0.64 to 2.45; *P* = .51), and *I*^2^ = 34%, suggesting low heterogeneity. Among them, 3 articles summarized REP. There was no significant difference in the odds of subsequent REP, with a pooled OR of 0.69 (95% CI: 0.09 to 5.55, *P* = .73), and *I*^2^ = 63%, suggesting moderate-to-high heterogeneity. The forest map is shown in Figure 3.

### 3.7. Risk of publication bias

Computer-based retrieval was used in this study, except for manual retrieval; meanwhile, a large number of studies excluded might have induced publication bias. In this study, the funnel plot was relatively symmetrical, indicating no obvious publication bias.

## 4. Discussion

EP requires an accurate and timely diagnosis. A delay in diagnosis may cause life-threatening bleeding due to rupture of the fallopian tube. Therefore, surgical intervention is considered the gold standard. In the 1980s, medical advances in imaging technology facilitated early diagnosis owing to better visualization, while people are now more concerned with the protection of fertility in the face of various treatment options.

### 4.1. Comparison of reproductive outcomes after MTX or surgery

This study found that fertility was significantly higher after MTX than after surgical treatment (OR = 1.52; 95% CI: 1.20 to 1.92), with similar adverse pregnancy rates (OR = 0.89; 95% CI: 0.47 to 1.69). The surgery in this study included salpingostomy and salpingectomy, the surgical level varied in different places, and the reproductive outcomes after surgery were related to surgical precision, proficiency, and skills. The forest map in this study suggested slight heterogeneity and the funnel plot indicated no obvious publication offset. The study population of the 20 cohort studies was more extensive, including 3530 individuals. The IUP rate was significantly higher after MTX treatment than after surgical treatment; however, adverse pregnancy outcomes did not differ between the 2 groups. Therefore, for patients with EP with stable hemodynamics and appropriate indications, MTX treatment should be prioritized to better protect future fertility.

### 4.2. Analysis of reproductive outcomes after MTX versus salpingostomy, MTX versus salpingectomy, salpingostomy versus salpingectomy

This study found that the reproductive outcomes of patients with EP after MTX were similar to those after salpingostomy. MTX led to higher fertility than salpingectomy, with IUP (OR = 2.11, 95% CI: 1.52 to 2.93). Salpingostomy led to higher fertility than salpingectomy, with IUP (OR = 1.61, 95% CI: 1.29 to 2.01). However, there were no differences in REP among the research groups. The heterogeneity analysis in this study showed low heterogeneity. A meta-analysis conducted by Meghan C. H al.^[30]^ included 2 RCT studies and 16 cohort studies. In RCT studies, there was no significant difference in the probability of subsequent IUP between patients undergoing salpingectomy and those undergoing salpingostomy (OR = 0.97, 95% CI: 0.71 to 1.33); however, the subjects in RCT studies were patients with normal fallopian tubes. In contrast, in the cohort study, the patients had a lower chance of intrauterine pregnancy after salpingectomy (OR = 0.45, 95% CI: 0.39 to 0.52), which was consistent with the findings of this study. The postoperative reproductive outcomes of EP are related to the surgical techniques. The wide application of minimally invasive techniques, continuous refinement of surgical techniques and improvements in salpingostomy are beneficial to the improvement of reproductive outcomes. In patients with EP, surgery itself increases the risk of infection and postoperative adhesions. Therefore, for women with decreased fertility requirements, if the activity is not high, MTX can be preferred to avoid surgical trauma and risks; when necessary, salpingostomy is recommended.

### 4.3. Analysis of reproductive outcomes after MTX versus expectant treatment

In this study, MTX was found to be similar to expectant treatment for fertility protection (OR = 1.25, 95% CI: 0.64 to 2.45). The REP was not significantly different between the 2 groups (OR = 0.69, 95% CI: 0.09 to 5.55). Expectant treatment was as safe and effective as MTX in EP patients with stable hemodynamics and decreased or lower levels of HCG. After treatment, fertility was similar, but MTX had more drug side effects. Baggio S et al^[25]^ compare reproductive outcome after surgical, MTX, and expectant management for tubal EP. They found that women successfully managed by expectation have better reproductive outcomes compared to those who underwent surgery, with the shortest time to achieve a subsequent intrauterine clinical pregnancy. Therefore, if safely applicable, the expectant management should be considered in the case of tubal EP.

### 4.4. The strengths and weaknesses

We implemented extensive search strategies and effective statistics synthesis. The quality of the included studies was evaluated according to the NOS scale. All the studies had higher scores on this scale, indicating a low risk of bias. The forests map suggested slight heterogeneity and the funnel plot indicated no obvious publication bias. However, 20 studies were included in this review, including only 4 RCT studies. More high-quality RCT are needed to improve article quality. Due to the large age range of the study subjects, it may be of more significance to divide the patients into 2 groups based on age: those under 35 years and those over 35 years to perform statistical analysis.

## 5. Conclusion

For hemodynamically stable patients with fertility requiring tubal EP, MTX treatment is non-inferior to expectant treatment but has advantages over surgery, especially salpingectomy, in improving natural pregnancy outcomes. In addition, salpingostomy is more advantageous than salpingectomy. Thus, ectopic pregnancy treatment should be individualized.

## 6. Other information

The review protocol can be accessed on inplasy.com.

The website of the international DOI Foundation, https://www.doi.org, then go to INPLASY.COM.

## Acknowledgments

The authors would like to thank all researchers in our research group.

## Author contributions

**Conceptualization:** Hong-Juan Hao, Li Feng.

**Data curation:** Hong-Juan Hao, Li-Fei Dong.

**Formal analysis:** Hong-Juan Hao, Xiao-Li Zhao.

**Investigation:** Hong-Juan Hao, Li Feng, Li-Fei Dong.

**Methodology:** Hong-Juan Hao.

**Project administration:** Hong-Juan Hao, Li Feng.

**Resources:** Hong-Juan Hao, Wei Zhang.

**Software:** Hong-Juan Hao, Wei Zhang.

**Supervision:** Li Feng.

**Validation:** Hong-Juan Hao.

**Visualization:** Hong-Juan Hao.

**Writing – original draft:** Hong-Juan Hao.

**Writing – review & editing:** Hong-Juan Hao, Li Feng.

## References

[1] Barnhart K Coutifaris C Esposito M. The pharmacology of methotrexate. Expert Opin Pharmacother. 2001;2:409–17. 11336595 10.1517/14656566.2.3.409

[2] Barnhart K van Mello NM Bourne T. Pregnancy of unknown location: a consensus statement of nomenclature, definitions, and outcome. Fertil Steril. 2011;95:857–66. 20947073 10.1016/j.fertnstert.2010.09.006 PMC3032825

[3] The American College of Obstetricians and Gynecologists, Committee on Practice Bulletins—Gynecology. ACOG Practice Bulletin No. 191: Tubal Ectopic Pregnancy. Obstet Gynecol. 2018;131:e65–77. 29232273 10.1097/AOG.0000000000002464

[4] Bouyer J Coste J Fernandez H. Sites of ectopic pregnancy: a 10-year population-based study of 1800 cases. Hum Reprod. 2002;17:3224–30. 12456628 10.1093/humrep/17.12.3224

[5] Mol BW Hajenius PJ Engelsbel S. Serum human chorionic gonadotropin measurement in the diagnosis of ectopic pregnancy when transvaginal sonography is inconclusive. Fertil Steril. 1998;70:972–81. 9806587 10.1016/s0015-0282(98)00278-7

[6] Forveille C Boulieu D. Ectopic pregnancy after in vitro fertilization: 6 cases. J Gynecol Obstet Biol Reprod (Paris). 1997;26:374–8. 9265062

[7] Naveed AK Anjum MU Hassan A. Methotrexate versus expectant management in ectopic pregnancy: a meta-analysis. Arch Gynecol Obstet. 2022;305:547–53. 34524502 10.1007/s00404-021-06236-y

[8] Barnhart KT Gosman G Ashby R. The medical management of ectopic pregnancy: a meta-analysis comparing “single dose” and “multidose” regimens. Obstet Gynecol. 2003;101:778–84. 12681886 10.1016/s0029-7844(02)03158-7

[9] Morlock RJ Lafata JE Eisenstein D. Cost-effectiveness of single-dose methotrexate compared with laparoscopic treatment of ectopic pregnancy. Obstet Gynecol. 2000;95:407–12. 10711553 10.1016/s0029-7844(99)00548-7

[10] Al Toqi A Al Shukri M Gowri V. Long term reproductive outcome following an ectopic pregnancy at a University Hospital. BJOG: Int J Obstet Gynaecol. 2021;128(SUPPL 2):23.

[11] Daniilidis A Panteleris N Chatzistamatiou K. Surgical and medical treatment in ectopic pregnancy: a retrospective analysis of 36 cases. Gynecol Surg. 2016;13:S279.

[12] Boychuk AV Khlibovska OI Yakymchuk YB. Ectopic pregnancy and its long-term results. Wiad Lek. 2020;73:139–44. 32124824

[13] Yousefnezhad A Pirdehghan A Roshandel Rad M. Comparison of the pregnancy outcomes between the medical and surgical treatments in tubal ectopi pregnancy. Int J Reprod Biomed. 2018;16:31–4. 29675485 PMC5899767

[14] Demirdag E Guler I Abay S. The impact of expectant management, systemic methotrexate and surgery on subsequent pregnancy outcomes in tubal ectopic pregnancy. Ir J Med Sci. 2017;186:387–92. 10.1007/s11845-016-1419-5 26895299

[15] Levin G Ohayon A Weissbach T. Ectopic first pregnancy treated by methotrexate versus salpingectomy – maternal and perinatal outcomes in a subsequent pregnancy: a retrospective study. Int J Gynecol Obstet. 2023; 160:823–8. 10.1002/ijgo.14365 PMC10087190 35871755

[16] Pereira GD Hajenius PJ Mol BWJ. Fertility outcome after systemic methotrexate and laparoscopic salpingostomy for tubal pregnancy. Lancet. 1999;353:724–5. 10.1016/s0140-6736(98)02250-8 10073522

[17] Fernandez H Capmas P Lucot JP. Fertility after ectopic pregnancy: the DEMETER randomized trial. Hum Reprod. 2013;28:1247–53. 23482340 10.1093/humrep/det037

[18] Bouyer J Job-Spira N Pouly JL. Fertility following radical, conservative-surgical or medical treatment for tubal pregnancy: A population-based study. Br J Obstet Gynaecol. 2000;107:714–21. 10.1111/j.1471-0528.2000.tb13330.x 10847225

[19] Olofsson JI Poromaa IS Ottander U. Clinical and pregnancy outcome following ectopic pregnancy; a prospective study comparing expectancy, surgery and systemic methotrexate treatment. Acta Obstet Gynecol Scand. 2001;80:744–9. 11531618 10.1034/j.1600-0412.2001.080008744.x

[20] Moeller LBK Moeller C Thomsen SG. Success and spontaneous pregnancy rates following systemic methotrexate versus laparoscopic surgery for tubal pregnancies: a randomized trial. Acta Obstet Gynecol Scand. 2009;88:1331–7. 19961341 10.3109/00016340903188912

[21] El-Sherbiny MT El-Gharieb IH Mera IM. Methotrexate versus laparoscopic surgery for the management of unruptured tubal pregnancy. Middle East Fertil Soc J. 2003;8:256–62.

[22] de Bennetot M Rabischong B Aublet-Cuvelier B. Fertility after tubal ectopic pregnancy: results of a population-based study. Fertil Steril. 2012;98:1271–6.e1. 22818285 10.1016/j.fertnstert.2012.06.041

[23] Dalkalitsis N Stefos T Kaponis A. Reproductive outcome in patients treated by oral methotrexate or laparoscopic salpingotomy for the management of tubal ectopic pregnancy. Clin Exp Obstet Gynecol. 2006;33:90–2. 16903244

[24] Düz SA. Fertility outcomes after medical and surgical management of tubal ectopic pregnancy. Acta Clin Croat. 2021;60:347–53. 10.20471/acc.2021.60.03.02 PMC8907964 35282476

[25] Baggio S Garzon S Russo A. Fertility and reproductive outcome after tubal ectopic pregnancy: comparison among methotrexate, surgery and expectant management. Arch Gynecol Obstet. 2021;303:259–68. 32852572 10.1007/s00404-020-05749-2 PMC7854461

[26] Turan V. Fertility outcomes subsequent to treatment of tubal ectopic pregnancy in younger Turkish women. J Pediatr Adolesc Gynecol. 2011;24:251–5. 21715197 10.1016/j.jpag.2010.12.007

[27] Alkhafajy WR Alyaseen FF. Tubal patency and pregnancy rate following surgical and medical treatments of ectopic pregnancy. Indian J Public Health Res Dev. 2018;9:1906–10.

[28] Shen YT Yang YY Zhang PG. Tubal ectopic pregnancy: a retrospective cohort study on clinical characteristics, treatment options and reproductive outcomes within 5 years. Arch Gynecol Obstet. 2022;306:2055–62. 36036288 10.1007/s00404-022-06690-2

[29] Asgari Z Chegini V Hosseini R. Fertility outcomes subsequent to medical and surgical treatment for ectopic pregnancy: a retrospective cohort study in Iran. Int J Reprod Biomed. 2021;19:881–8. 34805728 10.18502/ijrm.v19i10.9820 PMC8595907

[30] Ozcan MCH Wilson JR Frishman GN. A systematic review and meta-analysis of surgical treatment of ectopic pregnancy with salpingectomy versus salpingostomy. J Minim Invasive Gynecol. 2021;28:656–67. 33198948 10.1016/j.jmig.2020.10.014

